# Dietary intakes and daily distribution patterns of macronutrients in youth soccer players

**DOI:** 10.3389/fnut.2023.1134845

**Published:** 2023-04-20

**Authors:** Diogo V. Martinho, Robert J. Naughton, César Leão, João Lemos, Adam Field, Ana Faria, André Rebelo, Élvio R. Gouveia, Hugo Sarmento

**Affiliations:** ^1^Research Unit for Sport and Physical Activity, Faculty of Sport Sciences and Physical Education, University of Coimbra, Coimbra, Portugal; ^2^School of Human and Health Sciences, University of Huddersfield, Huddersfield, United Kingdom; ^3^Escola Superior de Desporto e Lazer, Instituto Politécnico de Viana do Castelo, Viana do Castelo, Portugal; ^4^Research Center in Sports Performance, Recreation, Innovation and Technology (SPRINT), Melgaço, Portugal; ^5^Manchester Metropolitan University, Manchester, United Kingdom; ^6^Polytechnic of Coimbra, Coimbra Health School, Dietetics and Nutrition, Coimbra, Portugal; ^7^Laboratory for Applied Health Research (LabinSaúde), Coimbra, Portugal; ^8^CIDEFES, Centro de Investigação em Desporto, Educação Física e Exercício e Saúde, Universidade Lusófona, Lisbon, Portugal; ^9^COD, Center of Sports Optimization, Sporting Clube de Portugal, Lisbon, Portugal; ^10^Department of Physical Education and Sport, University of Madeira, Funchal, Portugal; ^11^Laboratory of Robotics and Engineering Systems (LARSYS), Interactive Technologies Institute, Funchal, Portugal

**Keywords:** nutrition, carbohydrates, protein, energy expenditure, football

## Abstract

**Introduction:**

There has been an abundance of dietary analysis research conducted on adult male soccer players, while studies on youth players are lacking. Furthermore, the daily distribution of energy and macronutrient intake throughout the day has been reported to influence training adaptations, but this is often not considered in the literature. This study aims to quantify daily energy and macronutrient intake and assess their distribution over 5 days, and compare daily energy intakes and predicted daily energy expenditure in under-16 male soccer players.

**Methods:**

The sample included 25 soccer participants aged 14.8–15.7 years. Five-day self-reported food diaries were used to record the food/drink consumption. Intake was analyzed for total daily energy, macronutrient intakes, and distribution among meals (breakfast, lunch, dinner, and snacks). Daily energy expenditure was predicted by resting energy expenditure and physical activity levels developed for youth sports participants.

**Results:**

The mean total energy intake was 1,928 ± 388 kcal∙day^−1^, whereas the estimated daily energy expenditure was 3,568 kcal∙day^−1^. Relative daily protein intakes were lower at breakfast, morning snack, afternoon snack, and night snack compared to lunch and dinner.

**Discussion:**

Youth soccer players do not appear to meet energy requirements and daily CHO guidelines. Fluctuations in protein intake throughout the day were noted and may influence training adaptations (i.e., muscle protein synthesis and recovery).

## 1. Introduction

Soccer academies place significant demands on young players in order to facilitate their holistic development (1, 2). Analyses of external loads over the course of one season in the English Premier League (EPL) soccer academy found that adolescent players covered approximately 26.0 km p∙wk.^−1^, and the mean high-speed running distance was 657 and 749 m for under-15 and under-16 players, respectively (3). Indeed, the seasonal load indicators obtained in young soccer players were comparable to those reported in six elite adult players (4), particularly in the under-16 and under-18 age groups (3). Additionally, the reported total energy expenditure measured by accelerometry in under-16 players averaged 2,551 kcal∙day^−1^ (5), meaning that to support training and match loads, attention needs to be given to the nutritional recommendations in youth players, namely, energy intake, and the quantity, type, timing, and distribution of macronutrients. Previous studies in youth players have focused on total daily energy and macronutrient intakes (5–8), with findings indicating that recommended protein intakes were generally met, but energy and carbohydrate (CHO) requirements were not.

Studies focusing on daily energy intake in youth soccer players reported varied results. The mean energy intake using a 24-h dietary recall during four non-consecutive days in Dutch players was 2,938 kcal∙day^−1^ (9). The intake of Spanish players was assessed by 3-day food diaries, and higher values were obtained in three competitive age groups (14-year-olds: 3,456 kcal∙day^−1^; 15-year-olds: 3,148 kcal∙day^−1^; 16-year-olds: 3,478 kcal∙day^−1^) (6) compared to the Dutch sample. Daily energy intake was approximately 800 kcal∙day^−1^ lower than the predicted daily energy expenditure in Italian youth players (10). In addition, the mean daily energy intake estimated from self-reported food diaries in EPL academies in under-15 and under-16 players was 1,927 kcal∙day^−1^ (8), which may also indicate an insufficient intake in youth soccer participants. The consequences of negative energy balance (energy intake < energy expenditure) are associated with health problems (i.e., compromised bone health, reproductive function immunity, sub-optimal protein synthesis, increased risk of injury, and development of eating disorders) (11, 12).

The importance of CHO to fuel soccer training and competition and to promote glycogen replenishment is well documented (13). Moreover, the daily distribution of protein intake is essential to optimize the skeletal muscle adaptive response and enhance recovery (14–16), and consequently, an intake of 0.40–0.55 g∙kg^−1^∙meal^−1^ over at least four meals is recommended (17). Of note, pre-sleep protein intake potentiated changes in strength and body composition (18, 19) and improved hunger and appetite sensations (20). The distribution of energy intake throughout the day was related to total energy intake, with a higher energy intake in the morning being associated with a lower total energy intake (21). Studies on macronutrient distribution have focused on CHO periodization strategies according to training and match loads in adult male players (4, 13, 22). In youth players, a skewed distribution of protein intake was noted, which may be sub-optimal for training adaptations and recovery (8). However, this study combined intermediate meals and did not analyze variations in macronutrient intake across morning, afternoon, and evening snacks. In addition, information on daily energy intake and dietary patterns in Portuguese soccer academies is scarce.

Given the high physical demands placed on young soccer players, the purposes of this study were: (1) quantify total energy and macronutrient intakes; (2) compare total energy intakes with predicted energy expenditure and (3) examine the daily distribution of energy and macronutrient intakes. It was hypothesized that soccer players would not meet recommendations for energy and CHO intake, and that the distribution of protein would be skewed throughout the day.

## 2. Materials and methods

### 2.1. Ethical approval and procedures

The current study was approved by the Ethics Committee of the Instituto Politécnico de Coimbra (N.°56_CEIPC/2022) and followed the recommendations of the Declaration of Helsinki for research involving human subjects, prepared by the World Medical Association. Parents or legal guardians were informed about the nature, aims, and risks of the study and subsequently gave written informed consent. Soccer players were made aware that participation was voluntary and that they could withdraw from the study at any time.

### 2.2. Participants

Adolescent male soccer players (*n* = 25), aged 14.8–15.7 years, who were registered with a competitive club affiliated with the Portuguese Soccer Federation took part in this study. Participants completed four soccer training sessions per week and three strength and conditioning sessions per week under the supervision of a fitness coach. The average duration of the soccer and gym sessions was 90 and 45 min, respectively. Chronological age was calculated as the difference between the date of birth and the date of anthropometric assessment.

### 2.3. Anthropometry

Height, body mass, and skinfolds were measured by an experienced observer. Two skinfolds (triceps and calf) were measured to estimate the percentage of fat mass based on the following equation (23):


$$\%BF=0.735\;x\;\left(\mathrm{triceps}+\mathrm{calf}\right)+1.0$$

### 2.4. Dietary intake

Participants recorded each food item consumed for five consecutive days (four training days and one match day) during the season (November 2021) using a training diary. In athletes, 3–7 days are necessary to obtain accurate and precise estimates of habitual food intake (24–26). During this period, no nutritional intervention was implemented by the club in order not to influence food choices. A dietitian explained the instructions for completing the food diary. Supplements are usually consumed before, during, or post-training and may not be considered food by athletes (25); this point was previously explained to athletes and supplements should be included in the food diary. Time of consumption was used to categorize six meals: breakfast (meal consumed between 7:00–9:30 a.m.), morning snack (meal consumed between breakfast and lunch), lunch (meal consumed between 12:00–2:00 p.m.), afternoon snack (meal consumed between lunch and dinner), dinner (meal consumed after 9:30 p.m.), night snack (meal consumed between 11:30 p.m.–12:30 a.m.). Details of brand names, cooking and preparation methods, time of the meal, and the number of items ingested were obtained. In addition, players quantified the quantity of foods and fluids consumed by providing weight or volume details specific to the food package or using standardized household measures. The dietitian checked missing data and resolved problematic cases through individual interviews. Food diary records were analyzed using Nutritics software (version 3.74 professional edition, Nutritics Ltd., Co. Dublin, Ireland) by a single and expert observer to reduce variation in data interpretation (27). The main outputs extracted were overall total absolute, and relative to body mass, intakes of energy (kcal), CHO, protein, and fats and also considering the variation by meal.

### 2.5. Predicted total energy expenditure

Total energy expenditure was based on the Schofield-HW equation for estimating resting energy expenditure (28), which has been validated for male children and adolescents (29). Subsequently, the average physical activity level of 2.03 reported in male adolescent athletes was used to estimate total energy expenditure (30).

### 2.6. Statistical analysis

Descriptive statistics were calculated, and the normality of the distribution was checked using the Shapiro–Wilk test. Repeated measures of analysis of variance (ANOVA) with one factor tested the differences in energy and macronutrient intakes between meals. A limited number of players who consumed the night snack were therefore not considered in the analysis. The size of the effect was interpreted as follows (31): eta squared < 0.1 (trivial), 0.1 ≤ eta squared < 0.3 (small), 0.3 ≤ eta squared < 0.5 (moderate), 0.5 ≤ eta squared <0.7 (large), 0.7 ≤ eta squared <0.9 (very large), 0.9 ≤ eta squared (nearly perfect). *Post-hoc* comparisons with Bonferroni adjustment were used to identify differences between specific meals. Analyses were completed using SPSS for Windows (SPSS Inc., IBM Company, N.Y., United States) and GraphPad Prism (version 5.00 for Windows, GraphPad Software, San Diego California United States). Statistical significance was set at 0.05.

## 3. Results

**Figure 1:**
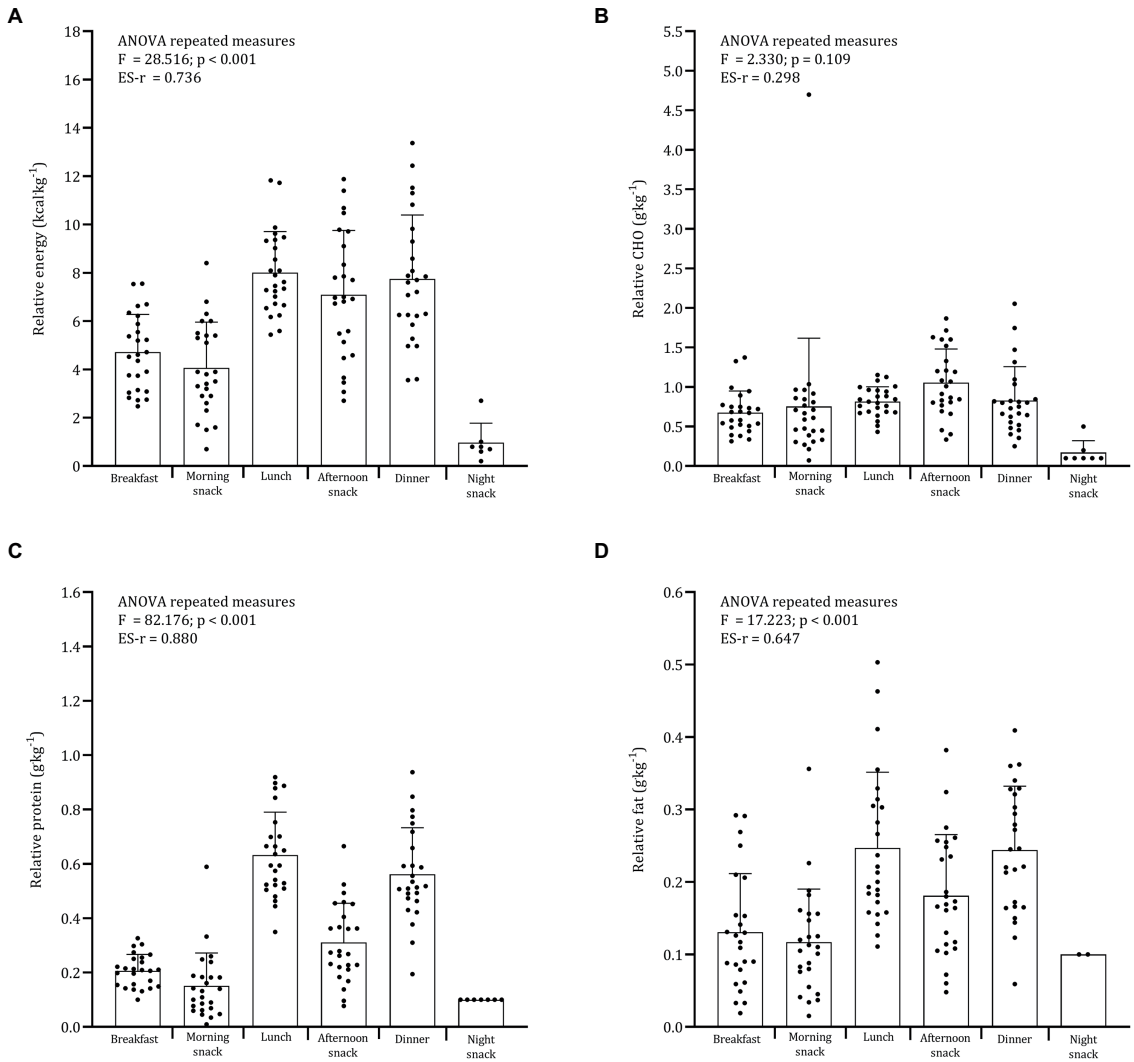
Daily distribution of relative **(A)** energy, **(B)** CHO, **(C)** protein, and **(D)** fat.

**Figure 2:**
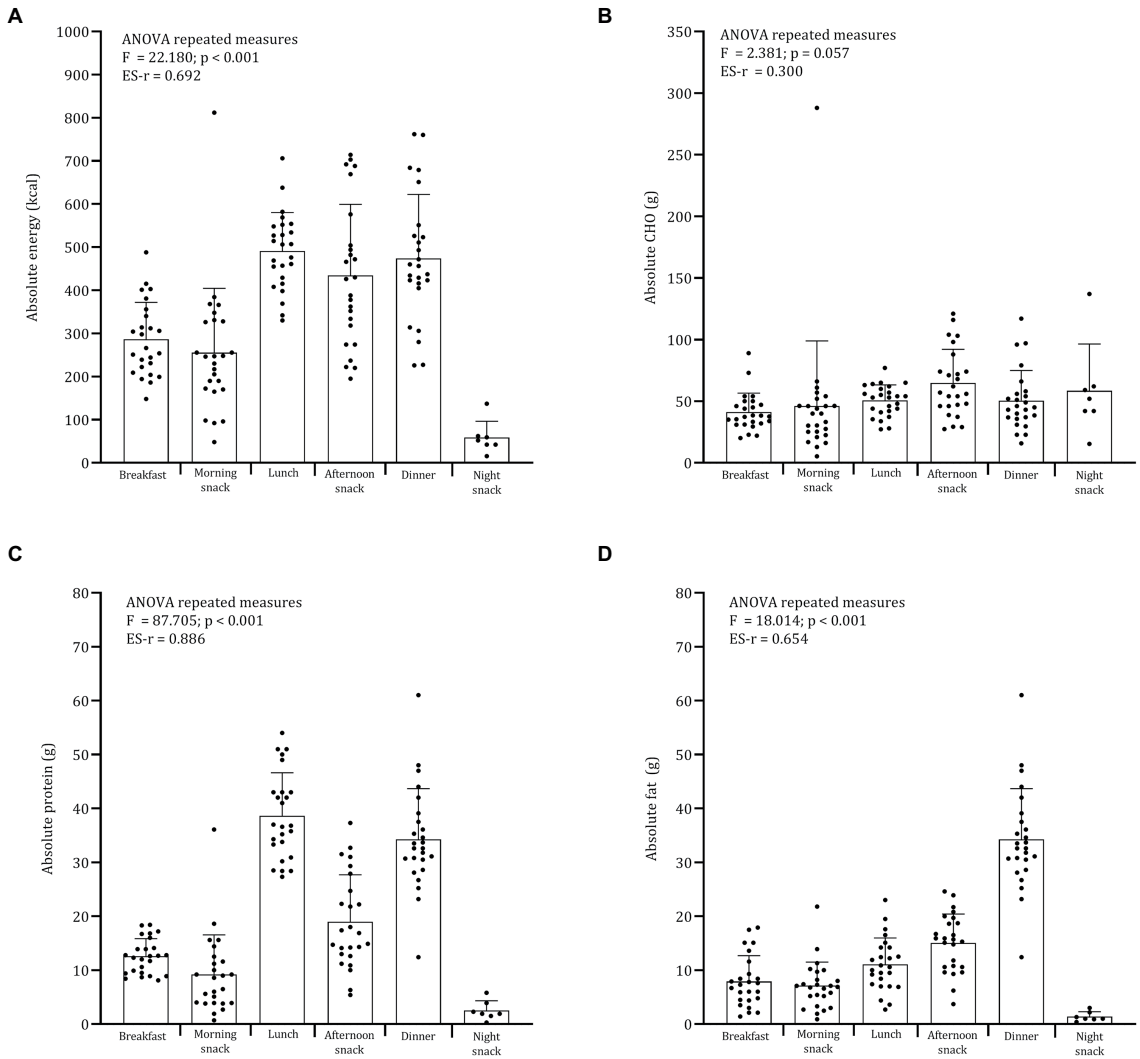
Daily distribution of absolute **(A)** energy, **(B)** CHO, **(C)** protein, and **(D)** fat.

Descriptive statistics for age, height, body mass, fat mass percentage, energy expenditure, and energy and macronutrient intake are summarized in Table 1. The mean estimated energy intake was, on average, 1,929 kcal∙day^−1^ while the predicted total energy expenditure was 3,568 kcal∙day^−1^. The adolescent soccer players ingested 4.0 g∙kg^−1^, 1.9 g∙kg^−1^, and 0.9 g∙kg^−1^ of CHO, proteins, and lipids, respectively. Table 2 shows the meal frequency for each day. The late snack is often not consumed by most players. Repeated measures ANOVA showed a significant difference in the distribution across meals for energy, protein, and fat intakes when expressed as absolute (Figure 1) or relative values (Figure 2). Energy intake was significantly lower at breakfast (286 kcal∙day^−1^) in comparison to lunch (491 kcal∙day^−1^), afternoon snack (434 kcal∙day^−1^), and dinner (474 kcal∙day^−1^). The average energy intake for the morning snack was 256 kcal∙day^−1^ and significant differences were noted with lunch, afternoon snack, and dinner.

**Table 1:** Characteristics, dietary intake and energy expenditure for the sample of male soccer players.

Variable	Units	Descriptive statistics			Normality	
		Range	Mean (95% CI)	Standard deviation	Shapiro–Wilk	*p*
Chronological age	years	(14.8; 15.7)	15.3 (15.2 to 15.5)	0.3	0.910	0.030
Height	cm	(156.0; 183.0)	171.1 (168.4 to 173.8)	6.5	0.950	0.251
Body mass	kg	(46.7; 81.4)	62.0 (59.0 to 65.0)	7.2	0.970	0.640
Fat mass	%	(8.2; 28.1)	16.1 (13.6 to 18.5)	5.9	0.194	0.016
Energy expenditure	kcal∙day ^−1^	(3,027; 4,232)	3,568 (3,565 to 3,672)	251	0.971	0.683
Absolute energy intake	kcal∙day ^−1^	(1,312; 2,842)	1929 (1768 to 2088)	388	0.968	0.583
Relative energy intake	kcal^.^kg^−1^	(20; 47)	32 (29 to 35)	7	0.962	0.460
Total carbohydrates	g	(149; 349)	245 (220 to 270)	61	0.939	0.142
Relative carbohydrates	g^.^kg^−1^	(2.4; 6.2)	4.0 (3.9 to 4.4)	1.0	0.959	0.393
Total protein	g	(79; 157)	114 (105 to 122)	22	0.966	0.547
Relative protein	g^.^kg^−1^	(0.9; 2.7)	1.9 (1.7 to 2.0)	0.4	0.985	0.963
Total fat	g	(33; 102)	55 (49 to 61)	15	0.912	0.034
Relative fat	g^.^kg^−1^	(0.6; 1.7)	0.9 (0.8 to 1.0)	0.2	0.902	0.021

**Table 2:** Frequency of meal intake per day.

Meal	Frequency of meals consumed (*n*)				
	TD1	TD2	TD3	TD4	MD
Breakfast	25	25	25	25	25
Morning snack	21	21	18	19	19
Lunch	25	24	25	25	24
Afternoon snack	23	25	25	23	25
Dinner	24	25	24	25	23
Night snack	2	2	2	4	0

TD (training day); MD (match day). *n* = 25.

Comparable results were found for relative energy intake. The distribution of CHO across meals was not significant whether expressed as absolute (*F* = 2.381, *p* = 0.057) or relative (*F* = 2.330, *p* = 0.109). Absolute and relative CHO intakes were significantly lower at breakfast (absolute: 41.2 g; relative: 0.68 g∙kg^−1^) than at the afternoon snack (absolute: 64.8 g; relative: 1.06 g∙kg^−1^). Absolute protein intakes were significantly lower at breakfast (12.6 g), morning snack (9.1 g), and afternoon snack (19.0 g) than at lunch (38.6 g) and dinner (34.2 g). Very large differences were found for relative protein intake across meals (*F* = 82.176; *p* < 0.001; ES-r = 0.880). For absolute (*F* = 18.014; p < 0.001; ES-r = 0.654) and relative intake (*F* = 17.223; *p* < 0.001; ES-r = 0.647) fat distribution, large differences were found across meals.

## 4. Discussion

The objectives of the current study were to compare the total energy intake with the predicted energy expenditure, to describe the daily macronutrient intakes, and to quantify the daily distribution of CHO, protein, and fat in a cross-sectional sample of youth male soccer players. First, energy intake was substantially lower in comparison to predicted energy expenditure; second, male soccer players did not meet recommendations for relative CHO intake; and finally, total energy intake and macronutrients presented an unbalanced distribution throughout the day. Given the demands of training and competition, the main findings of the present study have implications for soccer training adaptations, body composition, and pre-and post-training fueling.

Predicted daily energy expenditure based on the Schofield-HW equation to estimate resting energy expenditure and physical activity level of 2.03 reported in 23 youth athletes (30) was, on average, 3,568 kcal∙day^−1^. Lower values of daily energy expenditure using accelerometers (i.e., 2,550 kcal∙day^−1^) were found in 10 adolescent male soccer players (5), while energy expenditure derived from hours of soccer activity, body mass and thermic effects of macronutrients in 10 players was 3,618 kcal∙day^−1^ (7). More recently, mean energy expenditure measured by the doubly labeled water method over 14 days was 3,586, 3,029, and 2,589 kcal∙day^−1^ in under-18, under-15, and under-13 soccer players, respectively (32). Taking into account the differences between methods of estimating energy expenditure, a negative energy balance was consistent across studies with youth soccer players. A mean daily energy intake of 2,243 kcal∙day^−1^ using food diaries and 24-h recall methods was noted in the Premier League Soccer Academy, which corresponds to a mean daily energy deficit of −307 kcal∙day^−1^ (5). Another study that included players from the Premier League Soccer Academy also reported lower values of energy intake (under-12 and under-13: 2,659 kcal∙day^−1^; under-15: 2,821 kcal∙day^−1^; under-18: 3,180 kcal∙day^−1^) estimated by the remote food photography method compared to daily energy expenditure (under-12 and under-13: 2,859 kcal∙day^−1^; under-15: 3,029 kcal∙day^−1^; under-18: 3,586 kcal∙day^−1^) (32). A negative energy balance of 890 kcal∙day^−1^ was also found in 75 adolescent soccer players from junior teams of the Italian First Division Soccer League (10). The daily energy intake in the present study (i.e., 1,929 kcal∙day^−1^) was substantially lower than in the previous studies, which explains the highest energy deficit (i.e., −1,729 kcal∙day^−1^) found in the current sample. Nevertheless, a negative energy balance is related to the concept of low energy availability, which in turn has an impact on performance, bone health, reproductive function, immunity, protein synthesis, cardiovascular and mental health, increased risk of injury, and development of eating disorders (11, 33).

Low energy intake is partially associated with CHO intake in youth soccer players. CHO recommendations for young players to meet daily energy requirements are in the range of 6–8 g∙kg^−1^∙day^−1^ (32). In Spanish players under 17 years of age, a CHO intake of 5.39 g∙kg^−1^∙day^−1^ was reported (6), and 4.7 g∙kg^−1^∙day^−1^ of CHO intake was noted in under-15 and under-16 participants (8). The CHO intake in the present sample (i.e., 4.0 g∙kg^−1^∙day^−1^) was significantly lower than the recently proposed recommendations for youth soccer players (32). Taken together, these data suggest that youth soccer players do not meet current daily recommendations, which may negatively affect the demands imposed by training and match loads. In terms of protein guidelines, the protein requirements of adolescent soccer players based on the nitrogen balance method were 1.4–1.6 g∙kg^−1^∙day^−1^ (34, 35). These values are comparable to those recommended for adult participants (15, 17). The average relative daily protein intake in the present study was 1.9 g∙kg^−1^∙day^−1^, which was compared with previous data from Spanish (6), Italian (10), and English (8) soccer players. Soccer players tend to follow the requirements for daily protein consumption more than those for CHO. Consequently, in order to increase energy intake and achieve daily energy balance, Portuguese soccer academies should emphasize the relevance of CHO guidelines (type, timing, and quantity) for training and competition (36–38).

Recommendations for CHO intake 3–4 h before soccer training varied from 1 to 3 g∙kg^−1^ to ensure pre-exercise fueling. In addition, soccer players should achieve 1 g∙kg^−1^ of CHO per hour for 4 h in post-exercise (13). In the present sample, a normal distribution of CHO across meals was evident, and considering that players trained at 8:00 p.m. (between afternoon and dinner), the previous guidelines were not met, as shown in Figure 2. The mean CHO intakes of the meals before and after soccer training were 1.1 g∙kg^−1^ (afternoon snack) and 0.8 g∙kg^−1^ (dinner), respectively. Curiously enough, few players consumed the late snack, which is likely to have had a negative effect on the recovery process, especially considering the low CHO intake post-training. It must be noted that the guidelines were developed for adult soccer players and their application is limited for youth players. Lower levels of relative CHO intake were found at the breakfast (0.8 g∙kg^−1^). In seven EPL Academy soccer players (39), a 4.7% improvement in mean dribbling speed test was found when the players consumed an increased energy breakfast (497 kcal, 77 g CHO, 14 g protein, and 12 g fat) compared to a normal energy breakfast (268 kcal, 39 g CHO,10 g protein, and 8 g fat). In light of the above, a considerable amount of CHO should be consumed around training and breakfast.

The distribution of daily protein intake appears to play a crucial role in the modulation of muscle protein synthesis rather than the total daily protein intake (14, 40). In resistance-trained participants, the effect of doses of protein was examined under three different conditions 12 h post-exercise: eight servings of 10 g every 1.5 h; four servings of 20 g every 3 h; two servings of 40 g every 6 h. The highest rates of muscle protein synthesis were noted in athletes who consumed four servings of 20 g every 3 h (14). Similar results were found in 26 young active participants (40). Whole-body protein synthesis was higher with multiple doses of protein compared to a single dose (40). Recommendations for protein intake per meal/snack to optimize protein synthesis range from 0.22 to 0.33 g∙kg^−1^ every 3–4 h (18, 40). In the present sample of young male soccer players, the daily protein intake had a skewed distribution, which is consistent with recent data from EPL soccer players (8) and adult Dutch soccer players (41). Given these results, adjustments to daily protein intakes need to be considered, particularly at the night snack, breakfast, and morning snack. In addition, the mean protein intake at lunch and dinner was 0.6 g g∙kg^−1^ which may indicate that elevated amounts are being consumed. Of note, the frequency of night snacks was reduced over the 5 days. In fact, it has been shown that the ingestion of 40 g of casein protein 30 min immediately before bedtime increases amino acid availability, which in turn impacts muscle protein synthesis (42). The effects of CHO, slow (i.e., casein), and fast (i.e., whey) proteins on appetite and resting energy expenditure were studied in 11 active adult males. Although non-significant differences were found, satiety was greater in the protein groups compared to the CHO or placebo trials (43). The ingestion of a protein snack before bedtime should be encouraged in youth soccer players.

The present study has limitations that should be acknowledged. First, food diaries tend to under-report up to 20% of the total energy intake (44). Second, most of the macronutrient recommendations presented in this paper were based on adult soccer players (38). Nevertheless, guidelines for youth players are scarce. Total energy expenditure was predicted by equations so future studies need to quantify the energy expenditure using the doubly labeled water method in addition to training and match load. Finally, the sample is limited to a single Portuguese youth soccer team, therefore the generalizability of these results should be made with caution.

In conclusion, Portuguese adolescent soccer players did not meet the CHO recommendations. Daily protein intakes were, on average, met but the distribution of protein over the day had a significant fluctuation. Lower values of protein intake were reported during the night snack, breakfast, and morning snack, which has a negative impact on muscle protein synthesis. The low energy intake was associated with an average daily energy deficit, which is associated with health problems. Given the high demands of soccer training and matches, players should optimize their daily energy intake and follow the recommendations for CHO and protein intakes in terms of type, timing, and quantity.

## Data availability statement

The raw data supporting the conclusions of this article will be made available by the authors, without undue reservation.

## Ethics statement

The studies involving human participants were reviewed and approved by Instituto Politécnico de Coimbra. Written informed consent to participate in this study was provided by the participants’ legal guardian/next of kin.

## Author contributions

DVM, RN, AFa, and HS conceptualized and wrote the manuscript. DVM, CL, and JL collect and organized the data. DVM, AR, AFi, and ERG planned and performed the statistical analyses and figures. RN and AFi revised of the manuscript. All authors contributed to the article and approved the submitted version.

## Conflict of interest

The authors declare that the research was conducted in the absence of any commercial or financial relationships that could be construed as a potential conflict of interest.

## Publisher’s note

All claims expressed in this article are solely those of the authors and do not necessarily represent those of their affiliated organizations, or those of the publisher, the editors and the reviewers. Any product that may be evaluated in this article, or claim that may be made by its manufacturer, is not guaranteed or endorsed by the publisher.
